# Editorial: Nutrition and Physical Activity as Cornerstones of Women’s Health—Insights from the Special Issue “Nutrition, Physical Activity and Women’s Health”

**DOI:** 10.3390/nu17223531

**Published:** 2025-11-12

**Authors:** Małgorzata Mizgier, Xuewen Wang

**Affiliations:** 1 Department of Sports Dietetics, Faculty of Health Sciences, Poznan University of Physical Education, 61-871 Poznan, Poland; 2 Department of Exercise Science, School of Public Health, University of South Carolina, Columbia, SC 29208, USA; xwang@sc.edu

## 1. Introduction

Lifestyle behaviors such as nutrition and physical activity are fundamental determinants of women’s health across the lifespan. The interaction between diet physical activity, oxidative stress, inflammation, and hormonal regulation shapes not only reproductive outcomes but also long-term cardiometabolic and musculoskeletal health. Growing evidence supports that regular physical activity and optimal diet quality can attenuate oxidative stress and improve lipid metabolism and inflammatory profile in women. This integrative perspective is supported by the literature. Militello et al. (2024) highlighted that physical activity reduces oxidative stress in aging women by modulating redox signaling and antioxidant enzyme activity [1]. Onu et al. (2025) concluded that combining aerobic and resistance training with balanced nutrition significantly mitigates metabolic syndrome and systemic inflammation [2]. Complementing these findings, Porter et al. (2023) reported favorable lipoprotein remodeling in older women following exercise training, including increased HDL particle and LDL particle sizes, suggesting reduced cardiovascular risk even with moderate exercise doses [3]. Additionally, Mizgier et al. (2025) showed that oxidative stress, low-grade inflammation, and elevated androgens were associated with impaired bone metabolism in adolescent girls with polycystic ovary syndrome, underscoring the systemic impact of metabolic, inflammatory, and hormonal dysregulation on women’s reproductive and skeletal health [4].

Collectively, these studies underscore the essential role played by both diet and physical activity in maintaining oxidative, metabolic, and cardiovascular homeostasis in women, forming the scientific foundation for this Special Issue, entitled “Nutrition, Physical Activity and Women’s Health”, designed to foster a deeper understanding of how dietary and physical activity behaviors influence women’s health outcomes. The five included papers address diverse yet interconnected aspects—from adolescent athletes to older women—highlighting the multifaceted links between diet, exercise, hormonal regulation, inflammation, and body composition. Collectively, they provide evidence that lifestyle interventions are powerful tools for promoting women’s health across the lifespan.

## 2. Summary of Included Publications

### 2.1. Caffeine and Anaerobic Performance in Female Soccer Players

Mor et al. [5] examined the acute effects of caffeine (6 mg/kg) on anaerobic performance and functional strength in 13 female soccer players using a randomized, double-blind, placebo-controlled crossover design. Caffeine significantly improved minimum and average power during the Running-Based Anaerobic Sprint Test (RAST) and enhanced single-leg hop performance on the right side. No significant changes were observed in vertical jump, change in direction, handgrip, or ball speed. These findings confirm that caffeine supplementation can improve specific anaerobic performance parameters in female athletes and highlight the importance of ergogenic research for female athletes’ performance.

### 2.2. Water Intake, Dietary Acid Load, and Body Composition in Aging Females

Januszko and Białecka-Dębek [6] investigated the relationships between water intake, dietary acid load (PRAL), and body composition among 195 women aged 65–84 years. Higher water intake was associated with lower fat mass (total, android, and gynoid) and higher lean mass. Women with low-PRAL diets had higher appendicular lean mass compared to those with high-PRAL diets, and their diets were characterized by lower energy and protein intake but a higher plant-to-animal protein ratio. These results emphasize that hydration and acid–base balance may contribute to maintaining favorable body composition and muscle mass during aging.

### 2.3. Dietary, Hormonal, and Immuno-Metabolic Correlates of Primary Dysmenorrhea in Adolescent Basketball Players

Mizgier et al. [7] investigated differences in dietary habits, hormonal and immuno-metabolic parameters, and susceptibility to disordered eating attitudes (DEAs) between dysmenorrheic (D) and non-dysmenorrheic (no-D) young female basketball players. This study also aimed to identify risk factors for primary dysmenorrhea (PD), focusing on nutrition, anthropometric parameters, and biochemical markers. The D group showed higher EAT-26 scores and elevated prolactin and cortisol levels, which remained independent risk factors for PD in logistic regression analysis. These findings highlight the complex interplay between hormonal regulation, psychological stress, and eating behavior in adolescent athletes, suggesting the need for integrated lifestyle monitoring in sports practice.

### 2.4. Probiotic Supplementation and High-Intensity Interval Training in Primary Dysmenorrhea

Yang et al. [8] conducted a double-blind, randomized controlled trial evaluating the effects of probiotic supplementation and high-intensity interval training (HIIT) in women with PD. HIIT significantly reduced premenstrual symptoms, menstrual distress, and pain severity, alongside improvements in hormonal and inflammatory profiles (estradiol, prolactin, progesterone, cortisol, hs-CRP, prostaglandins). Probiotic supplementation reduced premenstrual and menstrual distress symptoms, though without significant effects on pain or hormonal markers. This study demonstrates that exercise is a potent non-pharmacological intervention for menstrual pain, while probiotics may offer additional support for symptom relief and improving quality of life.

### 2.5. Lifestyle Interventions in Polycystic Ovary Syndrome (PCOS): A Systematic Review

Gautam et al. [9] provided a comprehensive systematic review of lifestyle-based interventions in PCOS management. The review synthesized evidence from dietary, exercise, and behavioral studies published over the past decade. Across multiple intervention types, including energy restriction, low-glycemic diets, resistance and aerobic training, and combined lifestyle programs, consistent benefits were observed for insulin sensitivity, anthropometric outcomes, ovulatory function, and androgen profiles. These results reinforce lifestyle modification as the first-line treatment for PCOS and advocate for individualized, multidisciplinary approaches that integrate nutrition and exercise science into clinical practice.

## 3. Integrative Perspective

Taken together, the contributions to this Special Issue illustrate the multidimensional interconnections between nutrition, physical activity, and hormonal health. Whether in the context of dysmenorrhea, PCOS, or aging, women’s physiological responses to lifestyle factors reflect intricate interactions among metabolic, inflammatory, and endocrine systems. These studies collectively highlight three important insights, namely integration, personalization, and feasibility, suggesting that simple, evidence-based lifestyle strategies can meaningfully improve women’s health outcomes across the lifespan.

## 4. Emerging Directions

Future research should emphasize sex-specific experimental designs accounting for menstrual cycle phase and hormonal contraceptive use; longitudinal and mechanistic studies; integrated behavioral science approaches; and collaboration between researchers, clinicians, and policymakers to translate findings into practice. Such efforts will accelerate the transition toward precision lifestyle medicine for women’s health.

## 5. Conclusions

This Special Issue strengthens the evidence that nutrition and physical activity are essential to women’s physical and metabolic health. The included studies reveal new pathways through which lifestyle modulates performance, inflammation, and reproductive function, offering a foundation for personalized and preventive strategies that empower women throughout all stages of life.
